# Interstitial lung disease and pancreatic exocrine insufficiency in CADDS: Phenotypic expansion and literature review

**DOI:** 10.1002/jmd2.12390

**Published:** 2023-08-19

**Authors:** Oliver Heath, Dinusha Pandithan, James Pitt, Elena Savva, Laura Raiti, Jenny Bracken, Moya Vandeleur, Martin B. Delatycki, Joy Yaplito‐Lee, Winita Hardikar, Rebecca Halligan

**Affiliations:** ^1^ Department of Metabolic Medicine The Royal Children's Hospital Melbourne Australia; ^2^ Victorian Clinical Genetics Services Murdoch Children's Research Institute Melbourne Australia; ^3^ Department of Radiology The Royal Children's Hospital Melbourne Australia; ^4^ Department of Respiratory Medicine The Royal Children's Hospital Melbourne Australia; ^5^ Department of Gastroenterology The Royal Children's Hospital Melbourne Australia

**Keywords:** *ABCD1*, BAP31, *BCAP31*, CADSS, cholestasis, lungs, pancreas, peroxisomal

## Abstract

Contiguous *ABCD1*/ *DXS1357E* deletion syndrome (CADDS) is a rare deletion syndrome involving two contiguous genes on Xq28, *ABCD1* and *BCAP31* (formerly known as *DXS1357E*). Only nine individuals with this diagnosis have been reported in the medical literature to date. Intragenic loss‐of‐function variants in *BCAP31* cause the deafness, dystonia, and cerebral hypomyelination syndrome (DDCH). Isolated pathogenic intragenic variants in *ABCD1* are associated with the most common peroxisomal disorder, X‐linked adrenoleukodystrophy (X‐ALD), a single transporter deficiency, which in its more severe cerebral form is characterised by childhood‐onset neurodegeneration and high levels of very‐long‐chain fatty acids (VLCFA). While increased VLCFA levels also feature in CADDS, the few patients described to date all presented as neonates with a severe phenotype. Here we report a tenth individual with CADDS, a male infant with dysmorphic facial features who was diagnosed through ultra‐rapid whole genome sequencing (WGS) in the setting of persistent cholestatic liver disease, sensorineural hearing loss, hypotonia and growth failure and developmental delay. Biochemical studies showed elevated VLCFA and mildly reduced plasmalogens. He died at 7 months having developed pancreatic exocrine deficiency and interstitial lung disease, two features we propose to be possible extensions to the CADDS phenotype. We also review the genetic, phenotypic, and biochemical features in previously reported individuals with CADDS.

**Synopsis**

This paper describes a new patient with CADDS who developed pancreatic exocrine deficiency and interstitial lung disease. We summarise the genetic, phenotypic, and biochemical findings of all previously reported individuals with CADDS.

## 1 INTRODUCTION

Contiguous *ABCD1*/*DXS1357E* deletion syndrome (CADDS) is a rare deletion syndrome involving two contiguous genes that lie head‐to‐head on chromosome region Xq28, *ABCD1* and *BCAP31* (formerly known as *DXS1357E*).

*ABCD1* encodes the peroxisomal integral membrane ALD protein (ALDP) and belongs to the ATP‐binding cassette (ABC) transporter superfamily.^1^ Intragenic loss‐of‐function variants in *ABCD1* are associated with X‐linked adrenoleukodystrophy (X‐ALD; MIM#300100), a peroxisomal disorder and single enzyme (transporter) deficiency characterised by reduced ß‐oxidation of VLCFA, white matter demyelination and adrenal cortex atrophy. Elevated plasma VLCFA is present at birth in all affected individuals.^1^ Cerebral X‐ALD is the most severe form of the disorder characterised by childhood‐onset neurodegeneration from 3 years of age that, in the absence of bone marrow transplantation very early in the disease course, progresses to severe disability and death.^2^

*BCAP31* encodes B‐cell‐receptor associated protein 31 (BAP31), a ubiquitously expressed transmembrane protein located in the endoplasmic reticulum (ER), including in mitochondria‐associated membranes (MAMs).^3^ BAP31 functions both as a protein chaperone and quality control factor involved in ER export or retention, and ER‐associated degradation. It also acts as a MAM tetherer and regulatory protein, supporting mitochondrial homeostasis and regulating autophagy and apoptosis.^3^ Intragenic loss‐of‐function variants in *BCAP31* cause the deafness, dystonia and cerebral hypomyelination syndrome (DDCH; MIM#300475).^4^

CADDS was first described in 2002 by Corzo et al.,^5^ in three male infants with large *ABCD1* deletions extending into *BCAP31*. Since then, a further six patients with a similar phenotype and variable deletions involving these contiguous genes have been reported, including one symptomatic girl.^6^, ^7^, ^8^, ^9^ As in DDCH, severe developmental delay, dystonia, deafness, and white matter abnormalities are commonly reported, while the presence of raised VLCFA is like that seen in isolated *ABCD1* defects. However, unlike either DDCH or X‐ALD, CADDS affected males have persistent cholestatic liver disease and commonly die in infancy.^5^, ^6^, ^7^, ^8^, ^9^

Here, we describe a severely affected male infant with CADDS who was diagnosed through ultra‐rapid whole genome sequencing (WGS). We summarise the clinical and biochemical findings from all previously published cases of CADDS, while also suggesting that pancreatic insufficiency and interstitial lung disease may represent extensions to the phenotype.

## 2 CASE REPORT

The proband was a male infant with hepatic cholestasis, hypotonia, sensorineural hearing loss (SNHL), and failure to thrive referred for metabolic assessment at 4 weeks of age. He was the only child to non‐consanguineous Caucasian healthy parents and was born at term, small for gestational age (birthweight 2.44 kg, *Z* = −2.2) despite an otherwise uncomplicated pregnancy. He developed jaundice on day 2 with raised total bilirubin of 203 μmol/L (reference range: 0–15 μmol/L) and GGT of 1287 U/L (RR: 0–40 U/L). His stools remained acholic amidst persistent cholestatic liver dysfunction (Table 1). Failure to thrive was compounded by persistent feeding difficulties with poor suck and frequent emesis, together with ongoing malabsorption and steatorrhea due to pancreatic insufficiency (faecal elastase < 15 μg/g; Table 1).

**TABLE 1:** Proband's biochemical profile.

	Units	1 month*☨	3 months#	6 months^	RR
Liver function					
ALT	U/L	196	199	178	<50
ALP	U/L	290	626	418	100–350
GGT	U/L	267	585	644	0–40
INR	Ratio	1.1	2.7	1.1	0.8–1.2
Total bilirubin (% conjugated)	μmol/L	65 (66)	76 (73)	26 (62)	0–15
Fat‐soluble vitamins					
Vitamin A	μmol/L	1.3	<0.2	0.2	0.6–1.8
Vitamin D	nmol/L			15	50–160
Vitamin E	μmol/mmol	2	0.7	1.3	0.9–7.1
Exocrine/endocrine function					
Faecal elastase	μg/g	<15	<15	<15	>200
AM cortisol	nmol/L	191	268	552	100–440
ACTH	pmol/L		15	16.2	<20
Peroxisomal studies					
VLCFA					
C24/C22	ratio	1.727			0.550–1.050
C26/C22	ratio	0.144			0–0.03
Pristanate	μmol/L	0.01			0–2.5
Phytanate	μmol/L	0.9			0–20.0
RBC plasmalogens					
Plasmalogen C16:0/Hb	μg/g Hb	125			140–300
Plasmalogen C18:0/Hb	μg/g Hb	184			265–475
Bile acids					
Total plasma BA (fasting)	μmol/L	92		316	0–36
Urine taurocholic		+++	+++		
Urine THCA		++	++		

*Note*: Start of supplementation with ursodeoxycholic acid (*), fat‐soluble vitamins (☨), Creon (#) and cholic acids (^).

Abbreviations: ACTH, adrenocorticotropic hormone; ALP, alkaline phosphatase; ALT, alanine transaminase; BA, bile acids; Hb, haemoglobin; INR, international normalised ratio; RBC, red blood cell; RR, reference range; THCA, tauro tetrahydroxycholestanoic acid; VLCFA, very long chain fatty acids.

At 4 weeks of age, he appeared jaundiced and cachectic, his weight was 2.65 kg (*Z* = − 3.88) and head circumference 34.5 cm (*Z* = −2.20). He had triangular faces with a broad forehead and a pointed chin, and an occipital haemangioma that had remained unchanged since birth. His anterior fontanelle was not enlarged. He had central hypotonia with prominent head lag, but otherwise normal reflexes and anti‐gravity movements. There was no organomegaly. His vision was intact and auditory brainstem response audiometry confirmed bilateral moderate SNHL. Brain MRI at 4 weeks showed normal myelination and no evidence of cortical dysplasia, ischemia, or haemorrhage.

Tyrosinemia, galactosemia and Nieman Pick disease type C, together with more common causes of intra‐and extra‐hepatic cholestasis were excluded following initial investigations. Other monogenic differential diagnoses subsequently considered included peroxisomal disorders, disorders of bile acid metabolism, congenital disorders of glycosylation (e.g., ATP6AP1‐CDG), mitochondrial disorders and *JAG1* or *NOTCH2* defects.

## 3 METHODS

### 3.1 Biochemical and Fibroscan analyses

Pre‐treatment blood, urine and CSF samples were collected and processed for routine biochemistry, amino acids, organic acids, bile acids, VLCFA and plasmalogens according to standard protocols. Other urine metabolites were measured by flow‐injection tandem mass spectrometry using targeted multiple reaction monitoring as previously described.^10^ FibroScan™ was undertaken with the Echosens machine, S1 probe and 10 successful measurements.

### 3.2 Next‐generation sequencing analysis

Trio whole genome sequencing (WGS) was performed on DNA isolated from blood using massively parallel sequencing (Nextera™ DNA Flex Library Prep kit, Illumina Sequencers) with a mean target coverage of 30x and a minimum of 90% of bases sequenced to at least 10× for nuclear DNA (nDNA) and a minimum of 800× mean coverage for mitochondrial DNA (mtDNA). Data was processed, including read alignment to the reference genome (GRCh38) and to the revised Cambridge Reference Sequence (rCRS) mitochondrial genome (NC_012920.1). Variant calling was carried out using Cpipe or Mutect2 for nDNA and mtDNA, respectively.^11^ For nDNA, variant analysis and interpretation within the target region (RefSeq genes ±1 kb) was performed using Agilent Alissa Interpret and reported in accordance with HGVS nomenclature. Copy number variants were screened for using an internal detection tool, CxGo.^12^ Curation of nDNA variants was phenotype‐driven with custom gene and pre‐curated gene lists (https://panelapp.agha.umccr.org/) used for variant prioritisation. Classification of nDNA and mtDNA variants was based on ACMG guidelines.^13^

## 4 RESULTS

Ultra‐rapid, five‐day turnaround WGS identified a 67 kb de novo hemizygous deletion involving *ABCD1* and part of *BCAP31* on Xq28 (chrX: 153714363–153 781 647), that was orthogonally validated with chromosomal microarray. He had markedly abnormal peroxisomal studies, with raised very long chain fatty acids and reduced plasmalogens (Table 1). He was nutritionally supported with a medium chain triglyceride‐based formula. Pancreatic exocrine insufficiency was managed with Creon and fat‐soluble vitamin supplementation. Cholestatic treatment with ursodeoxycholic acid (10 mg/kg BD) was commenced from 4 weeks of age with limited effect.

**FIGURE 1:**
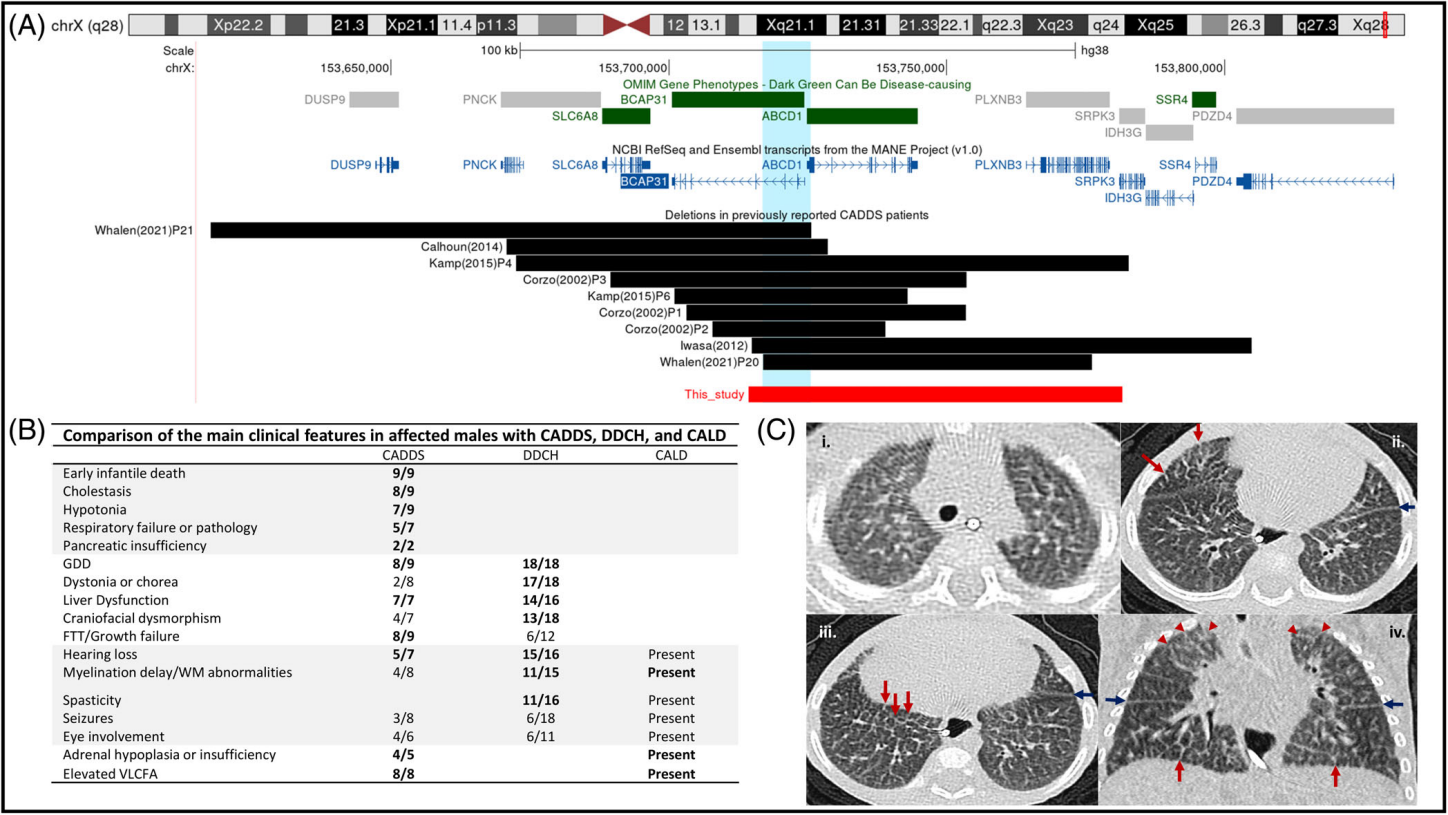
Schematic representation of features in CADDS patients reported to date. (A) Location and size of the chr Xq28 deletions (UCSC Genome Browser, GRCh38). Deletions are depicted with black (previous cases) and red (current case) bars, and the 11.7 kb region of overlap highlighted in blue. The exact 5′ breakpoint in van de Kamp et al.'s^8^ P6 is unknown. (B) Comparison of the main clinical features in hemizygous males with CADDS, deafness, dystonia, and cerebral hypomyelination syndrome (DCCH) or cerebral X‐linked adrenoleukodystrophy (CALD). DCCH data relates to patients with intragenic loss‐of‐function variants in *BCAP31* as reported in Whalen et al.^9^ The most prominent features are highlighted in bold. (C) Unenhanced CT chest at 6 months, lung windows. Axial images in the upper (i), mid (ii), lower zones (iii) and coronal image (iv) show diffuse smooth interlobular septal thickening (red arrows) and smooth interlobar fissural thickening (blue arrows) bilaterally. In addition, patchy ground glass opacity is seen bilaterally, especially in the upper zones (red arrowheads).

At 6 months, he was hospitalised for nutritional and respiratory assessments. His weight had stagnated (weight 3.55 kg, *Z* = −7.20), necessitating a transition to continuous nasojejunal feeds because of recurrent vomiting. He had global developmental delay with profound hypotonia and poor head control. He had stridor and increased work of breathing and lung CT showed unexplained diffuse interstitial opacities (Figure 1C), without specific features of aspiration pneumonitis and unremarkable infective indices. Liver stiffness measurement measured by Fibroscan was 12.9 kPa. This would be consistent with significant fibrosis; however, interpretation is limited due to the size of the patient and the lack of well‐established normal values in infants under 5 kg. Hepatic ultrasound at 1 month old showed normal echogenicity. Due to ongoing cholestasis a trial of oral cholic acid (15 mg/kg/day) was commenced, but he died at 7 months, before the effect of this medication could be ascertained.

## 5 DISCUSSION

Contiguous *ABCD1*/*DXS1357E* deletion syndrome (CADDS) is an ultra‐rare condition with only 10 individuals reported, including this patient. Their genetic, phenotypic, and biochemical characteristics are summarised in Table 2.

**TABLE 2:** Clinical, biochemical, and molecular features of CADDS.

Reference	Whalen P21	Kamp P4	Calhoun	Corzo P3	Kamp P6	Corzo P1	Corzo P2	Iwasa	Whalen P20	This report	Total
Molecular											
Deletion size	108 kb	110 kb	58 kb	64 kb	34–42 kb	50 kb	31 kb	90 kb	60 kb	67 kb	
Involved genes	*DUSP9*, *PNCK*, *SLC6A8*, *BCAP31*, *ABCD1*	*PNCK*, *SLC6A8*, *BCAP31*, *ABCD1*, *PLXNB3*, *SRPK3*	*SLC6A8*, *BCAP31*, *ABCD1*	*SLC6A8*, *BCAP31*, *ABCD1*	*BCAP31*, *ABCD1*	*BCAP31*, *ABCD1*	*BCAP31*, *ABCD1*	*BCAP31*, *ABCD1*, *PLXNB3*, *SRPK3*, *IDH3G*, *SSR4*, *PDZ4*	*BCAP31*, *ABCD1*, *PLXNB3*	*BCAP31*, *ABCD1*, *PLXNB3*, *SRPK3*	
Inheritance	nd	nd	Maternal	De novo	nd	Maternal	Maternal	De novo	De novo	De novo	
Clinical											
Sex	F	M	M	M	M	M	M	M	M	M	9M:1F
Age	3 year	Died <5 month	Died 11 month	Died 4 month	Died 8 month	Died 11 month	Died 4 month	Died 8 month	Died 16 month	Died 7 month	Died: 9
Cause of death		nd	LF	LF, RF	nd	LF, GI bleeding	RF, GI bleeding	Pneumonia, sepsis	RF, lung infiltrates	RF, lung infiltrates	
Growth											
IUGR/SGA	−	nd	+	+	−	−	−	+	+	+	5/9
FTT	−	nd	+	+	+	+	+	+	+	+	8/9
Developmental											
GDD	+	nd	+	+	+	+	+	+	+	+	9/9
GI											
Cholestasis	+	+	+	+	+	+	+	+	−	+	9/10
Liver dysfunction	nd	nd	+	+	nd	+	+	+	+	+	7/7
Pancr. insufficiency	−	nd	nd	nd	nd	nd	nd	nd	+	+	2/3
Hepatomegaly	−	nd	+	nd	nd	nd	nd	+	−	−	2/5
Splenomegaly	−	nd	+	nd	nd	nd	nd	−	−	−	1/5
Neurological											
Hypotonia	−	−	+	+	+	+	+	+	−	+	7/10
Seizures	−	nd	+	+	−	−	+	−	−	−	3/9
Dystonia/chorea	−	nd	−	−	−	+	−	−	+	−	2/9
Myelination delay	+	+	−	+	−	nd	+	+	−	−	5/9
SNHL	+	+	−	nd	nd	+	+	+	−	+	6/8
Ophthalmological	+	nd	+	nd	−	+	nd	+	+	−	5/7
Endocrine											
Adrenal hypoplasia/insufficiency	nd	nd	+	+	nd	nd	nd	+	+	−	4/5
Craniofacial/skin											
Dysmorphism	−	nd	+	−	+	−	−	+	nd	+	4/8
Haemangiomas	−	−	+	−	−	−	−	−	−	+	2/10
Biochemical											
VLCFA	nd	Elevated	Elevated	Elevated	Elevated	Elevated	Elevated	Elevated	nd	Elevated	
Plasmalogens	nd	nd	nd	Normal	nd	Normal	Normal	Normal	nd	Low	
Phytanic acid	nd	nd	nd	Normal	nd	Normal	Normal	Normal	nd	Normal	
Plasma L‐pipecolic	nd	nd	nd	Normal	nd	Normal	Normal	nd	nd	nd	
Urine organic acids	nd	nd	nd	Normal	Normal	nd	nd	Dicarboxylic p‐OH‐phenylacetate	nd	Mild dicarboxylic aciduria	
Bile acids											
Plasma	nd	nd	nd	Elevated	nd	nd	Elevated	nd	nd	Elevated	
Urine	nd	nd	nd	−	nd	nd	−	nd	nd	Elevated taurocholic	

*Note*: Patient order is based on the location of their deletion (centromeric to telomeric).

Abbreviations: +, presence of feature; −, absence of feature; F, female; FTT, failure to thrive; GDD, global developmental delay; GI, gastrointestinal; IUGR, intrauterine growth restriction; LF; liver failure; M, male; nd, no data; Pancr, pancreatic; RF, respiratory failure; SGA, small for gestational age; SNHL, sensorineural hearing loss; VLCFA, very long chain fatty acids.

In the current genomic era, the increased clinical availability of NGS platforms has greatly contributed to the discovery of rare diseases, also shortening diagnostic odysseys, and allowing earlier implementation of relevant management and/or surveillance measures. The CADDS diagnosis in our patient was established in an intensive care setting through access to ultra‐rapid turnaround trio WGS. This avoided the need for a liver biopsy and other invasive procedures during the patient's diagnostic workup, reflecting the clinical utility of rapid WGS in decreasing infant morbidity.^14^, ^15^ We acknowledge that this deletion would have been identified by microarray, albeit at a slower turnaround time, and that in general, performing microarray before genomic sequencing should be the norm unless there is particular urgency to go straight to WES/WGS.

The Xq28 deletions reported in CADDS vary in length between 31 and 110 kb (Figure 1A). The critical region common to all deletions will become better delineated as more affected individuals are identified. The deletions reported to date all span an 11.7 kb area that includes the promoter regions of both genes, the first exon of *ABCD1* and exons 1–3 of *BCAP31* (Figure 1A). RNA analyses and immunocytochemical studies on cultured fibroblasts from affected CADDS patients have previously confirmed the absence of *BCAP31* transcripts and ALDP (the protein encoded by *ABCD1*), respectively, in keeping with a loss‐of‐function effect.^5^, ^8^

Our proband's phenotype is consistent with previous reports, where most individuals presented in early infancy with hepatic cholestasis, severe growth failure, profound hypotonia, developmental delay and elevated VLCFAs (Table 2).^5^, ^6^, ^7^, ^8^, ^9^ SNHL occurred in 6/8, while cerebral hypomyelination and white matter changes were described in 5/9. Early death in hemizygous males (3–16 months) is common, although a symptomatic 3‐year‐old girl with CADDS is the longest surviving individual reported.^8^ The milder phenotype in this female is likely due to her having an unaffected X chromosome.

The presence of additional genes encompassed by separate deletions makes comparison between patients with CADDS challenging, given the possible contributions of each gene to the overall phenotype. Four affected individuals had deletions involving the creatine transporter gene *SLC6A8*. Delayed myelination (3/4 vs. 2/5) and seizures (2/3 vs. 1/6) were more frequently reported in this group compared to those whose deletions did not span *SLC6A8* (Table 2), reminiscent of features also seen in cerebral creatine deficiency syndrome 1 (CCDS1; MIM#300352). The deletion in our proband includes two other genes currently without gene‐disease associations. *PLXNB3* encodes plexin B3 which functions as a receptor for the semaphorin‐5A molecule and plays a role in axon guidance, invasive growth, and cell migration.^16^ There has been recent speculation about its involvement in cardiogenesis and neurodevelopment through Notch signalling regulation.^17^ However, congenital heart disease did not feature in any of the CADDS patients with *PLXNB3* involvement.^6^, ^8^, ^9^ *SRPK3* encodes a protein member of the serine/arginine‐rich specific kinase family that is known to be involved in mRNA processing, and synaptic vesicle and neurotransmitter release.^18^ A zebrafish knockout study in which *Srpk3* ^−/−^ fish were raised to adulthood has recently revealed *SRPK3* to be a compelling candidate gene for X‐linked intellectual disability.^19^ The impact of its deletion in our patient and two others is unclear, however, due to the early mortality reported in CADDS.^6^, ^8^

The separate contributions of *BCAP31* and *ABCD1* to the CADDS phenotype can only be partially gleaned from disorders caused by isolated deficiencies of these genes, DDCH and X‐ALD, respectively (Figure 1B). Spasticity, dystonia, chorea, deafness and cerebral hypomyelination appear to feature more prominently in individuals with intragenic loss‐of‐function variants in *BCAP31*. Neither DDCH nor X‐ALD, can account for the neonatal cholestasis and early infantile death underpinning the severity in CADDS. Rather, the combined loss of *BCAP31* and *ABCD1* has been suggested to exert a synergistic deleterious effect on bile acid synthesis,^9^ possibly accounting for the hepatic cholestasis in this condition.^7^, ^8^ Similar synergistic effects are implicated in the lethality of CADDS.^7^ Our patient died at 7 months of age having developed pancreatic insufficiency and unexplained lung infiltrates (Figure 1C). Similar features were reported in another male infant with a 60 kb deletion including *BCAP31*, *ABCD1*, and *PLXNB3* who died of respiratory failure at 16 months,^9^ and overall, death from respiratory causes is reported in 50% of males with CADDS.^5^, ^6^ Pancreatic insufficiency and interstitial lung disease are therefore possible extensions to the CADDS phenotype (Figure 1B).

Although the underlying pathophysiology in CADDS remains unclear, further exploration into the role of BAP31 in driving the severity of the phenotype is warranted, given the pleiotropic function of this protein. Concomitant loss of *BCAP31* has been suggested to exacerbate the clinical phenotype of *SLC6A8* deficiency, with patients exhibiting more pronounced hypotonia and developmental delays than expected, together with severe failure to thrive, dystonia and choreoathetosis.^8^, ^20^, ^21^ Similar effects are possibly at play in contiguous *ABCD1* and *BCAP31* deletions. Interestingly, BAP31 has been postulated to be involved in the cystic fibrosis disease process, as it targets CFTRdelta508 for ER associated degradation despite any residual transport activity that the partially misfolded CFTR protein may retain.^3^ The cholestatic liver disease and pancreatic insufficiency in our proband are reminiscent of features also seen in cystic fibrosis. Those similarities, however, do not extend to the respiratory findings identified on CT, which showed a diffuse interstitial process rather than bronchiectasis and air‐trapping. BAP31 also interacts with the bile salt export pump BSEP,^3^ and its involvement in mitochondrial homeostasis offer alternative avenues of exploration into its possible roles in the aetiology of cholestatic liver disease in CADDS. Unfortunately, we were not able to explore these hypotheses further, as our patient died before an adequate sweat test could be collected or fibroblast cultures established.

Oral cholic acid is an efficacious therapy in bile acid synthesis disorders and adjunctive treatment of peroxisomal disorders in patients who exhibit manifestations of liver disease, steatorrhea, or complications from decreased fat‐soluble vitamin absorption without liver fibrosis.^22^ Unfortunately, our patient died before the effects of this medication could be ascertained. His plasmalogens were mildly reduced and urine bile acid intermediates such as tetrahydroxycholestanoic (THCA) remained raised in normal proportion to other markers of liver dysfunction (Table 1), making an underlying peroxisomal biogenesis disorder unlikely. Previous studies, have reported normal plasmalogen levels in blood and cultured fibroblasts, with normal peroxisomal size, number and assembly demonstrated in liver tissue by electron microscopy and immunochemistry.^5^, ^6^ *BCAP31* deficiency potentially exacerbates the peroxisomal phenotype in CADDS, although the mechanisms remain unclear.^6^

## 6 CONCLUSION

This report adds to the limited body of knowledge that currently exists about CADDS, although many questions remain regarding the underlying pathophysiology of this condition. More investigations are needed to understand the synergistic effect of combined *ABCD1* and *BCAP31* loss of function. Pancreatic insufficiency and interstitial lung disease are possible extensions to the CADDS phenotype, while further biochemical characterisation of affected patients will be important to elucidate the extent to which peroxisome metabolism is compromised in this condition. Finally, ultra‐rapid genomic sequencing can help shorten patients’ diagnostic odysseys, potentially precluding the need for invasive procedures.

## AUTHOR CONTRIBUTIONS

**Oliver Heath, Dinusha Pandithan, Laura Raiti, Moya Vandeleur, Martin B. Delatycki, Joy Yaplito‐Lee, Winita Hardikar, Rebecca Halligan:** Clinical patient care and diagnosis. **Oliver Heath, Dinusha Pandithan, Laura Raiti, James Pitt, Elena Savva, Jenny Bracken, Moya Vandeleur, Martin B. Delatycki, Joy Yaplito‐Lee, Winita Hardikar, Rebecca Halligan:** Evaluation of metabolic, genetic, and radiologic results. **Oliver Heath, Rebecca Halligan:** Planning of the manuscript. **Oliver Heath:** Drafting of the manuscript. **Oliver Heath, Dinusha Pandithan, Laura Raiti, James Pitt, Elena Savva, Jenny Bracken, Moya Vandeleur, Martin B. Delatycki, Joy Yaplito‐Lee, Winita Hardikar, Rebecca Halligan:** Revision of the manuscript.

## CONFLICT OF INTEREST STATEMENT

Oliver Heath, Dinusha Pandithan, James Pitt, Elena Savva, Laura Raiti, Martin B Delatycki, Jenny Bracken, Moya Vandeleur, Joy Yaplito‐Lee, Winita Hardikar and Rebecca Halligan have approved the manuscript and declare that they have no conflict of interest. They did not receive reimbursements/fees/funds/salaries from an organization that may in any way gain or lose financially from the results reported in the reviewed manuscript in the last 5 years and have no other competing financial or non‐financial interests, as outlined in the JIMD Conflict of Interest form.

## AKNOWLEDGEMENTS

The authors thank the patient's family for their participation.

## INFORMED CONSENT

All procedures followed were in accordance with the Helsinki Declaration of 1975, as revised in 2000. Written informed consent was obtained from the patient's parents for collection of samples and publication of medical data.

## ETHICAL APPROVAL

Not required.

## Data Availability

This manuscript has no associated data.
